# Interactive Conversational Agents to Improve Dietary Behaviors for Health Promotion: Mixed Systematic Review

**DOI:** 10.2196/78220

**Published:** 2025-11-28

**Authors:** Samira Amil, Marie-Pierre Gagnon, Alexandra Bédard, Sié Mathieu Aymar Romaric Da, Daniela Zavala Mora, Vicky Drapeau, Sophie Desroches

**Affiliations:** 1 Centre Nutrition, santé et société (NUTRISS) – INAF, Université Laval, Pavillon des Services, 2440, boulevard Hochelaga, Office 2729-P, Quebec, QC, G1V 0A6, Canada, 1 418 656-2131 ext. 405564; 2 School of Nutrition, Université Laval, Quebec, QC, Canada; 3 VITAM – Centre de recherche en santé durable, Université Laval, Quebec, QC, Canada; 4 Centre de recherche du CHU de Québec, Université Laval, Quebec, QC, Canada; 5 Faculty of Nursing, Université Laval, Quebec, QC, Canada; 6 Bibliothèque, Direction des services conseils, Université Laval, Quebec, QC, Canada; 7 Département de kinésiologie, Faculté de médecine, Université Laval, Quebec, QC, Canada

**Keywords:** conversational agents, chatbots, computer, natural language processing, systematic review, dietary behaviors, chronic diseases prevention, health promotion, general population, artificial intelligence

## Abstract

**Background:**

Chronic diseases are the leading global cause of death, largely driven by Western lifestyles characterized by poor diets and physical inactivity. Digital interventions offer promising tools to support health behavior change. Interactive conversational agents (CAs) provide real-time, personalized meal planning and dietary advice. Their interactive nature and adaptability make them valuable for promoting healthy dietary behaviors in the context of diet-related chronic diseases. However, evidence of their effectiveness remains limited. Systematic evaluations of their impact, features, and user acceptability are needed to clarify their role in public health strategies for improving dietary behaviors and preventing chronic diseases.

**Objective:**

This review aimed to evaluate the effectiveness of CAs in improving dietary behaviors, to describe their features, functions, conversational capabilities, and impact on nutritional knowledge, usability, acceptability, user experience, and engagement.

**Methods:**

Five electronic databases were searched: MEDLINE, CINAHL, Embase, Web of Science, and PsycINFO. We only included sources that focused on the use of CAs to change dietary behavior. Eligible studies were published since 2013 in English, French, or Spanish. Two independent reviewers screened studies, with a third resolving disagreements. The quality of studies was appraised using the Mixed Methods Appraisal Tool (McGill University). Quantitative and qualitative findings were synthesized narratively.

**Results:**

In total, 2200 references were identified, and after screening and eligibility assessment, 11 references (10 studies with approximately 20‐480 participants) were included. Among the included studies, improvements in fruit and vegetable intake were reported in 2 studies (*P*=.04 and *P*=.005). One study found significant increases in adherence to the Mediterranean diet at 6 weeks with gains maintained at 12 weeks. Two additional studies reported enhanced nutritional knowledge (eg, nutrition label use). Effects on protein, whole grains, sugar, sodium, and caffeine intake were mixed or nonsignificant. Some studies reported increased physical activity (+109.8 min/wk) and reduced alcohol use for stress management. One randomized controlled trial showed modest but significant weight loss and decrease in waist circumference (−2.1 cm, 95% CI −3.5 to 0.7; *P*=.003). Engagement varied between studies. Usability and user experience were generally positive; goal setting, feedback, and tailored recommendations were linked to higher satisfaction. Reported challenges included unnatural conversation style, simplistic content, and limited perceived usefulness.

**Conclusions:**

CAs show promising potential to improve dietary behaviors, with evidence of gains in fruit and vegetable intake, Mediterranean diet adherence, nutritional knowledge, physical activity, and modest weight loss. Overall usability was favorable, but variability in outcomes, high attrition, and limited impact on social support highlight areas for refinement. Future research should use larger samples, longer follow-up, standardized outcomes, and strategies to enhance sustained engagement and inclusivity. Systematic evaluations and refined designs are essential to establish the role of CAs as scalable, evidence-based tools in chronic disease prevention.

## Introduction

Chronic diseases, including cardiovascular disease, diabetes, dementia, and cancer, are the world’s leading cause of death, accounting for approximately 74% of all-cause deaths [1]. This alarming situation has led to chronic diseases being identified as a major priority for action by the United Nations and the World Health Organization [1].

The etiology of chronic diseases is complex and multifactorial, and the current epidemic is largely attributed to a nutritional transition marked by the adoption of Western lifestyles. This transition, characterized by lower-quality diets, sedentary lifestyles, smoking, and excessive alcohol consumption, is evident in both high-income and low-income countries [2]. In response to these challenges, public health experts underscore the significance of preventive interventions at individual and population levels to reverse these trends and promote well-being [3]. However, current preventive and digital health interventions face important limitations. Many digital programs rely on static content delivery, lack personalization, and often fail to sustain user engagement over time. Barriers such as digital literacy, limited interactivity, and reduced adherence further restrict their long-term effectiveness [4,5]. These gaps create opportunities for innovative approaches. Conversational agents (CAs), through their interactive and adaptive design, can address these limitations by providing real-time feedback, personalized guidance, and continuous engagement, thereby offering a potentially more effective tool for promoting sustainable dietary behavior change [6-8].

In the digital age, between 20% and 80% of people use the internet to monitor their health and access a variety of resources [9]. Therefore, digital interventions offer promising ways to change lifestyle-related behaviors, particularly dietary ones. These interventions are defined as products or services that use computer technology to promote behavior change. They can be accessed through various media, including handheld devices, digital platforms, and smartphone apps [10].

Previous research [11-16] has shown that in addition to their possible effect on dietary behaviors, these interventions can lead to significant changes in several areas of health, including weight management [11], smoking cessation [12,13], increased physical activity (PA) [14], reduced alcohol consumption [15], or self-management of chronic diseases [16]. Their effectiveness often depends on engagement and ongoing interaction with the target population [17].

Several types of digital interventions have already been used to improve dietary behavior. mHealth apps provide tools for self-monitoring and goal setting, while web-based platforms deliver structured educational programs and personalized feedback [18,19]. Wearable devices have also been used to track dietary intake and PA, thereby supporting behavior changes [20]. While these tools can deliver short-term improvements, they are often limited by a lack of personalization and interactivity, resulting in declining user engagement over time [21]. CAs, in contrast, offer a dynamic, interactive interface that simulates human dialogue, providing real-time, context-sensitive guidance and maintaining ongoing engagement. These gaps create opportunities for an innovative approach [6-8,22,23].

Based on artificial intelligence, CAs simulate human conversations and provide personalized support to users. Their ability to provide real-time information, adapt to individual needs, and maintain ongoing engagement makes them particularly interesting for health interventions [24]. In the field of nutrition, chatbots could play a crucial role in providing personalized nutritional advice, helping with meal planning, answering nutrition-related questions, and encouraging healthy eating habits. Their 24/7 availability and ability to process large amounts of information make them potentially powerful tools for supporting lasting changes in dietary behaviors [25]. However, despite their apparent potential, the true effectiveness of CAs in improving dietary behaviors and preventing diet-related chronic diseases remains largely unexplored. Systematic evaluation of their impact, features, and user acceptability is crucial to determining their value in public health strategies to promote the adoption of healthy dietary behaviors and ultimately prevent diet-related chronic diseases [26].

To our knowledge, no systematic review has specifically examined the effectiveness of chatbots on dietary behaviors in the general population.

The primary objective of this mixed systematic review is to assess the effectiveness of interactive CAs designed to improve dietary behaviors. Secondary objectives are to list their basic features, functions, and conversational capabilities and, where possible, also assess their impact on nutritional knowledge, as well as their usability, acceptability, user experience, and engagement.

## Methods

### Overview

The review was structured using the Preferred Reporting Items for Systematic Reviews (PRISMA) guidelines (Checklist 1) [27]. To ensure transparency and rigor, a completed flow diagram was included in accordance with the PRISMA recommendations.

The systematic review was conducted in several stages: a comprehensive literature search, meticulous study selection, detailed data extraction, rigorous quality assessment, thorough data analysis, and synthesis of findings [28]. The protocol for this review was registered with the International Prospective Register of Systematic Reviews (PROSPERO) under the identifier CRD42023458561.

### Eligibility Criteria

This review adheres to clearly defined eligibility criteria, guided by the Population, Intervention, Comparison, Outcomes, Study Design (PICOS) framework, to ensure a focused and reproducible selection of relevant studies, enhancing the rigor of the systematic review process. The population of interest is the general population, regardless of age, to maximize the scope and generalizability of the findings. The intervention focuses on interactive CAs delivered via any available interactive digital platform, such as smartphones, web applications, or other digital devices, specifically designed to improve dietary behaviors. Both comparator and no comparator studies were considered to ensure inclusivity and to capture a wide range of evidence. The primary outcome of interest is the effect of CAs on dietary behaviors, while secondary outcomes include assessments of nutrition knowledge, usability, acceptability, engagement, and user experience. To leverage the complementary strengths of different research approaches, we included quantitative, qualitative, and mixed methods studies in this review to provide a holistic understanding of the impact of CAs on dietary behavior change [29].

We included studies published since 2013 in English, French, or Spanish. This timeframe was chosen based on the findings of Lyzwinski et al [30], who reported that 96% of publications on the use of chatbots for lifestyle behavior change were published after 2013. Languages were chosen because they are predominant in scientific literature, and the research team members are fluent in these languages. Dissertations, theses, and relevant websites of recognized nutrition-related organizations were not included in the review due to time limitations and insufficient human resources. We excluded syntheses of knowledge such as systematic reviews, editorials, opinion pieces, conference abstracts, and commentaries.

### Search Strategy

The search strategy was developed in collaboration with a librarian (DZM) and is detailed in Multimedia Appendix 1. This strategy was implemented in 5 electronic bibliographic databases: MEDLINE, CINAHL, Embase, Web of Science, and PsycINFO. The search covered literature published between January 2013 and December 2024. The initial database search was conducted on October 16, 2023, and updated on December 17, 2024. In addition, the reference lists of relevant papers were reviewed to ensure the inclusion of all eligible studies.

### Data Collection and Analysis

#### Study Selection

We conducted the review using the online platform Covidence systematic review software (Veritas Health Innovation Ltd). We imported all references into the tool, and most duplicates were removed automatically. Three reviewers (SA, SMARD, and AB) independently assessed the abstracts and titles of the studies identified by the search strategy after removing duplicates. Relevant studies were selected according to the predefined inclusion criteria. Following this stage, three independent reviewers (SA, SMARD, and AB) screened the full texts to identify eligible studies. A fourth, senior reviewer (SD) was consulted to resolve any disagreements.

#### Data Extraction and Management

Two reviewers (SA and SMARD) independently extracted data from the included studies. The following information was extracted using an extraction grid: general information (title, authors, country of study, funding, and year of publication); study details (aim, design, inclusion and exclusion criteria, method of randomization, and allocation); study population (age, sex, sample size, and number for analysis); intervention characteristics (type, duration, follow-up points, chatbot name, broadcast platform, language, and interactivity); and outcomes (primary and secondary outcomes, and method of outcome assessment). Disagreements were resolved by consensus or by consultation with a third reviewer. Authors were contacted in case of missing information or ambiguity.

#### Assessment of Quality in Included Studies

After the data extraction step, 2 independent reviewers assessed the study quality of the included studies using the Mixed Methods Appraisal Tool (MMAT; McGill University) [31]. In case of disagreement, the reviewers discussed the matter before consulting a third reviewer.

The MMAT assesses the methodological quality of 5 study categories (qualitative, randomized, nonrandomized, quantitative descriptive, and mixed methods). Each study is appraised against 5 criteria, rated as “yes,” “no,” or “can’t tell.” According to the MMAT guidelines, calculating a single overall score is not recommended. However, consistent with common practice in systematic reviews, we summarized the proportion of criteria met and presented the results as stars (from 1 star=20% of criteria met to 5 stars=100%). Higher ratings indicate stronger methodological quality.

#### Data Synthesis and Analysis

We conducted a descriptive analysis to summarize the extracted data and provide a narrative synthesis of the findings. This included a breakdown of results by subgroups such as age, gender, ethnicity, and geographical location of the population. Through the data synthesis, we described the effectiveness of current CAs; identified key features, functions, and conversational capabilities of successful chatbots; assessed their usability, acceptability, engagement, and user experience; and highlighted limitations and future research directions.

A meta-analysis was not planned because the study designs, populations, interventions, and outcome measures were too different from each other to allow meaningful quantitative pooling of the data.

### Deviations From the Registered Protocol

Initially, we decided to exclude conference papers, but during the selection process, we realized that some conference abstracts were showing results. We have included 1 conference abstract [32]. Despite the initial intention to explore gray literature sources, including dissertations, theses, and relevant organizational websites, this endeavor was not realized due to time constraints and limited human resources.

## Results

### Study Selection

**Figure 1.**
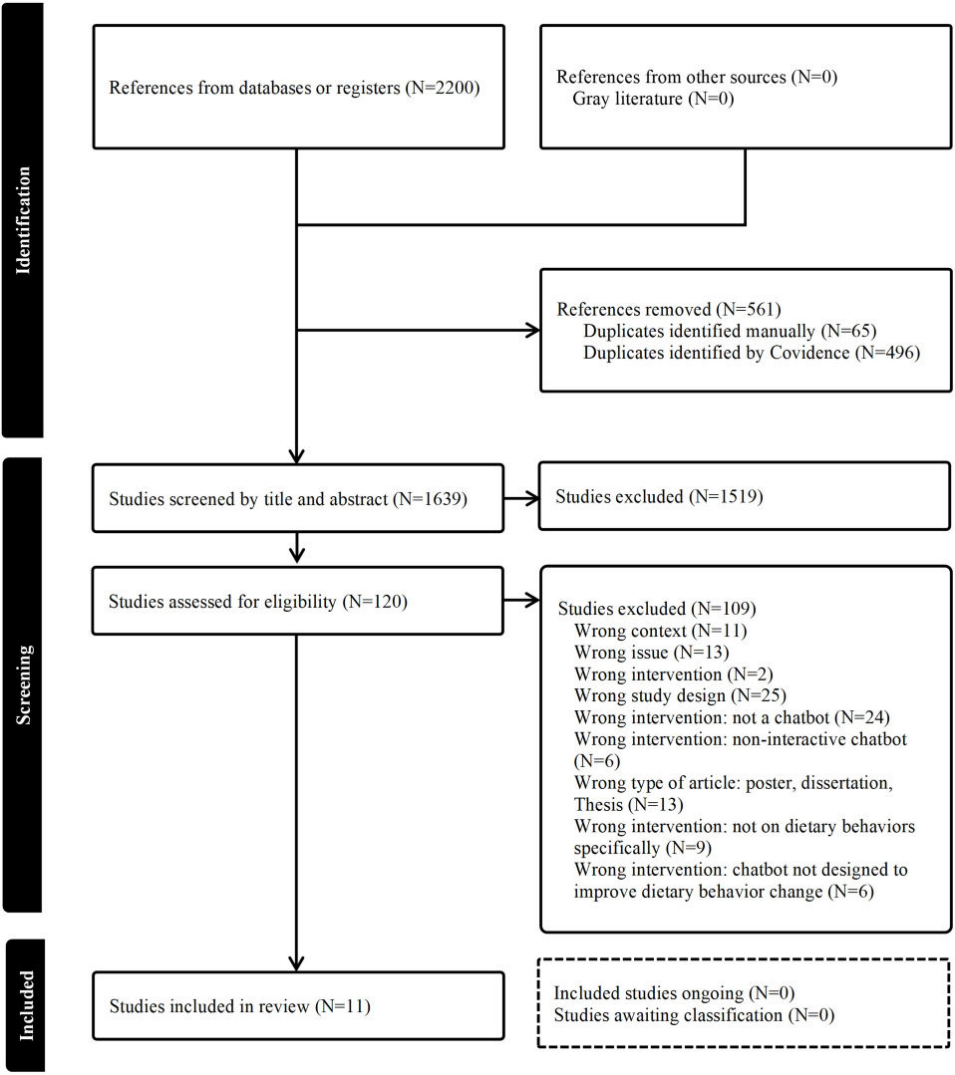
A Preferred Reporting Items for Systematic Reviews (PRISMA) flow diagram of literature search for the included studies.

The first search of the electronic databases was performed in November 2023 (1619 citations) and was followed by an update in December 2024 (581 citations). These 2 searches yielded 2200 citations in total. After 561 duplicates were removed, the titles and abstracts of the remaining 1639 studies were screened, excluding 1519 studies. The remaining 120 studies were read in full and excluded if applicable, leaving a final set of 11 studies. The detailed process of study selection is presented in the PRISMA flow diagram (Figure 1).

### General Characteristics of the Included Studies

The characteristics of the selected studies are summarized in Table S1 in Multimedia Appendix 2 [22,32-41]. The 11 included papers were published between 2013 and 2024 with one of these published in 2024 [33], 5 in 2022 [22,32,34,35], 1 in 2021 [36], 1 in 2020 [37,38], 2 in 2017 [39,40], and 1 in 2013 [41].

All studies were conducted in high-income countries: the United States (5/11, 46%), the Netherlands (2/11, 18%), Australia (1/11, 9%), the United Kingdom (1/11, 9%), Singapore (1/11, 9%), and the Republic of Korea (1/11, 9%). Most of the studies (6/11, 55%) were randomized trials. The remaining studies incorporated a combination of qualitative (2/11, 18%), mixed methods (1/11, 9%), and pre-post (2/11, 18%) studies. The sample size of the 10 studies that provided this information ranged from 20 to 480 participants. Almost all the studies (10/11, 91%) were performed among adults (mean age ranging from 15.0, SD 0.7 y to 73, SD 5.33 y) while 1 study was conducted among adolescents (mean age of 15.0, SD 0.7 y).

Most of the reviewed interventions (9/11, 82%) included more females than males, and the ethnicity of the included studies that disclosed this characteristic of participants was mainly from the Caucasian (White) or Chinese populations. Four of the studies (4/11, 36%) included participants with obesity and/or overweight as a health condition.

All studies focused on interventions that primarily aimed to change dietary behaviors through CA. The duration of these interventions varied from a single interaction to 12 months. CAs used in interventions are more frequently embodied CAs (7/11, 64%) rather than rule-based CAs (4/11, 36%). Embodied CAs are computer-generated animated humanlike characters that interact with users through verbal and nonverbal behavioral cues [42]. Rule-based CAs match the user input to a rule pattern and select a predefined answer from a set of responses with the use of pattern-matching algorithms [43]. The majority of these CAs (8/11, 73%) used avatars, seven of which were female, and one of which allowed users to choose the ethnicity of the avatar.

The interaction patterns of these agents were predominantly text-based (10/11, 91%), with some incorporating voice interactions, figures, images, or video links. A minority (1/11, 9%) used buttons or drop-down lists for predefined responses. The deployment of these agents occurred via digital platforms (7/11, 64%) or social networking applications (3/11, 27%) such as Facebook Messenger or Slack. Several theoretical models and techniques were used in the development of these CAs such as the transtheoretical model (TTM; 7/11, 64%), social cognitive theory (2/11, 18%), self-determination theory (SDT; 1/11, 9%), fuzzy trace theory (1/11, 9%), motivational interviewing (3/11, 27%), shared decision-making (1/11, 9%), persuasion techniques (1/11, 9%), nudging techniques (1/11, 9%), and feedback and rewards (1/11, 9%).

### Outcomes of the Included Studies

#### Dietary Behavior Effects

##### Overview

This review of studies examining the impact of CAs on dietary behaviors reveals a paucity of consistent results across various outcomes (Table S2 in Multimedia Appendix 2 [22,32-41]).

##### Consumption or Intake

Studies (4/11, 36%) examining dietary intake have shown varied outcomes across different food groups and intervention approaches. Regarding fruits and vegetables, Bickmore et al [41] found that participants in the diet group consumed significantly more daily servings compared to the control group. The combined intervention (PA+diet) also led to improvements, though the diet-only intervention yielded the best results (*P*=.005). Gardiner et al [40] reported a significant increase in fruit consumption among users of CAs compared to the control group participants (μ=2-4 vs μ=2-2; *P*=.04), although changes in vegetable intake were not statistically significant. In contrast, Kramer et al [34] found that CAs were unable to persuade users to modify their fruit and vegetable intake. Maher et al [38] demonstrated notable improvements in adherence to a Mediterranean-style diet, including fruits and vegetables, at 6 weeks, with these changes maintained at 12 weeks (mean change from baseline to 12 wk: 5.7; 95% CI 4.2‐7.3; *P*<.001).

Findings related to protein consumption were less consistent (3/11, 27%). Brust-Renck et al [39] observed no significant differences between the intervention group and the control group in the weekly consumption of proteins such as fish or red meat. Similarly, Gardiner et al [40] reported no notable changes between groups. While Maher et al [38] identified initial improvements in healthy protein intake as part of Mediterranean diet adherence, participants faced difficulties meeting recommended servings by week 6.

For whole grain intake (3/11, 27%), Brust-Renck et al [39] and Gardiner et al [40] both reported no meaningful differences between the intervention group and the control group. Maher et al [38] noted improved adherence to Mediterranean diet guidelines, including whole grains, but participants expressed challenges in maintaining the recommended intake.

Other dietary intakes yielded mixed results (4/11, 36%). Saravanan et al [32] observed no significant differences between experimental groups regarding calorie or sugar reduction, but 86% of participants across conditions achieved their goals. Brust-Renck et al [39] found no significant changes in sugar and sodium intake as well as other dietary intakes between the intervention and the control groups. Lee et al [33] reported a significant 60% decrease in weekly sugar intake from beverages while sodium and caffeine consumption from carbonated and energy drinks did not decrease. Kramer et al [34] reported that CA interventions did not influence liquid intake.

##### Eating Behaviors

Eating behaviors are defined here as a “normal behavior related to eating habits, selecting foods that you eat; culinary preparations and quantities of ingestion” [44]. In our review, all interventions aiming to improve eating behaviors yielded mixed results, with some studies highlighting limited impact and others demonstrating moderate success (4/11, 36%). Gardiner et al [40] found that 69% of participants used CA suggestions to improve eating habits such as consumption of fruits and vegetables, soda, caffeine, snacks, whole grains, red meat, and fish, but this was not significantly different from those relying on patient information sheets (66%). Kramer et al [34] identified a significant correlation between competence and eating behaviors (*r=–*0.38*; P=*.03), with competence also predicting eating behavioral changes over time (*F*_1.30_*=*4.30*; P=*.047*; R*²*=*0.13). Pecune et al [36] noted that 47% of CA users accepted healthy recipe recommendations, which were healthier than their initial preferences. While participants tended to opt for healthier recipes when the CA included explanations with its recommendations, the results did not reach statistical significance. Finally, Smriti et al [35] emphasized the influence of parents on their children’s eating habits, as improved parental feeding behaviors positively shaped those of their children. CAs facilitated reflection and supported families, though challenges related to complex family dynamics were noted. Overall, these findings suggest that while CAs can moderately influence eating behaviors, barriers such as family factors need to be addressed.

##### Behavioral Intentions

Only 2 (18%) studies examined behavioral intentions. Brust-Renck et al [39] reported that behavioral intentions were correlated with self-reported adoption of a healthy diet (weekly consumption of fruits and vegetables, fish, whole grains, sugar, and sodium). Declared intentions regarding nutrition were also linked to participants’ understanding and adherence to fundamental dietary recommendations. In contrast, Dhinagaran et al [22] reported that participants expressed no intention of changing their lifestyle, reflecting a lack of readiness to engage with the intervention.

##### Self-Efficacy

Gardiner et al [40] reported no significant differences in self-confidence in eating healthily between participants who used CAs and those relying on patient information sheets.

##### Adherence to Dietary Recommendations

Studies evaluating adherence to dietary recommendations (3/11, 27%) provide insights into how CAs can influence dietary behaviors. While adherence was mentioned in 3 studies [38,39,41], only Maher et al [38] explicitly reported measurable improvements.

Maher et al [38] focused on adherence to the Mediterranean diet, demonstrating significant improvements over a 12-week intervention led by a virtual health coach named Paola. Participants increased their Mediterranean diet adherence scores, maintaining these changes throughout the study.

Bickmore et al [41] incorporated dietary recommendations from the National Institutes of Health and the National Cancer Institute into their intervention. These guidelines were used to encourage increased fruit and vegetable consumption and to set personalized dietary goals. However, the study did not report specific data measuring participants’ adherence to these recommendations.

Brust-Renck et al [39] used American Heart Association (AHA) dietary recommendations to design their intervention, emphasizing gist comprehension to promote heart-healthy behaviors. However, adherence to AHA recommendations was not directly measured, making it difficult to assess the effectiveness of the intervention in changing long-term behaviors.

### Main Characteristics of CAs

Across the included studies, 2 main categories of CAs were identified: embodied CAs (ECAs) and rule-based CAs (RBCAs). ECAs (7/11, 64%) were the most common and primarily relied on text-based chat (6/11, 55%), with some incorporating voice-based interactions (4/11, 36%). These were typically deployed through web platforms (6/11, 55%) or dedicated applications (eg, Slack; 1/11, 9%) and frequently featured female avatars (5/11, 46%). In some cases, both male and female avatars (1/11, 9%) were available, or avatars were culturally adapted (eg, African American avatars, 2/11, 18% or avatars representing 3 different ethnicities, 1/11, 9%). In contrast, RBCAs (4/11, 36%) relied on structured interactions through predefined options such as buttons, drop-down menus (1/11, 9%), or scripted responses (1/11, 9%), and were integrated into social media platforms (eg, Facebook Messenger: 2/11, 18% or web-based interfaces: 1/11, 9.1%), often with minimal representations (eg, a robot head: 1/11, 9% or no avatar at all: 1/11, 9%).

From a theoretical perspective, both ECAs and RBCAs commonly drew upon the TTM (7/11, 64%) and social cognitive theory (2/11, 18%), while some incorporated additional frameworks such as SDT (1/11, 9%), fuzzy-trace theory (1/11, 9%), motivational interviewing (3/11, 27%), and shared decision-making (1/11, 9%). Techniques including persuasion (1/11, 9%), nudging (1/11, 9%), feedback (1/11, 9%), and rewards (1/11, 9%) were also reported as strategies to promote engagement and support behavior change. While text-based interactions predominated, some interventions included voice features (4/11, 36%) and visual elements (2/11, 18%) such as figures, images, or videos. However, 2 (18%) studies provided limited or unclear specifications regarding the agents’ modalities (1/11, 9%) [36] or theoretical underpinnings (1/11, 9%) [22], reflecting heterogeneity in design and implementation.

### Secondary Outcomes

#### Overview

In addition to the dietary behaviors–related primary outcomes discussed earlier, a review of secondary outcomes from included studies revealed a complex yet promising landscape (Table S3 in Multimedia Appendix 2 [22,32-41]).

#### Nutritional Knowledge

Nutritional knowledge has improved significantly in several studies (5/11, 46%). Brust-Renck et al [39] reported enhanced knowledge about energy balance, food labels, fast food, and advertising, with healthier self-reported dietary behaviors associated with greater nutrition knowledge. The study highlighted that users who actively engaged with the tutorial demonstrated a better understanding of the AHA dietary principles. Lee et al [33] found that awareness of nutrition labels increased from 64.3% to 92.9%, and nonreaders of nutrition labels decreased from 42.9% to 16.7%. Dhinagaran et al [22] highlighted positive feedback on diabetes prevention content, which was considered detailed and informative. However, participants who were already familiar with healthy living found the content less relevant.

#### Other Lifestyle Behaviors

PA outcomes were reported in 2 (18%) of 11 studies. Bickmore et al [41] found that participants in the PA group increased daily walking more rapidly than the control group, although no significant differences in International Physical Activity Questionnaire scores were observed. Brust-Renck et al [39] noted improvements in PA-related knowledge and self-reported behaviors. Significant increases in weekly PA were reported by Maher et al [38], with gains of 109.8 minutes over 12 weeks.

Stress management was another area of focus. Dhinagaran et al [22] highlighted the positive effects of CAs in promoting relaxation techniques, such as mindfulness, while Gardiner et al [40] reported a significant reduction in alcohol use for stress relief among the CA intervention participants compared to the control group (*P*=.03).

#### Social Support

The impact of CAs on social support outcomes was observed in 1 (9%) study. Kramer et al [34] observed no reduction in loneliness among participants, indicating limited influence in this domain.

#### Motivation

Several studies (3/11, 27%) reported positive effects on motivation to engage in behavior change. Gardiner et al [37] found significant progress in advancing stages of change at 6 months, particularly in behaviors related to diet and supplementation, though these effects diminished at 12 months. Saravanan et al [32] demonstrated that a memory model, which enables social CAs to recall and reference past interactions to deliver personalized, motivational dialogues based on users’ progress and emotions, significantly boosted motivation, with the greatest increases observed in specific experimental conditions created to evaluate the effects of memory references and run the experiment as a between-subjects design. Smriti et al [35] noted that CAs encouraged parents to reflect on their eating habits, motivating them to adopt healthier behaviors that benefit both themselves and their children.

#### Engagement

User engagement with CAs is less well documented, and this engagement varies between studies (5/11, 46%). Gardiner et al [37,40] reported a median duration of interactive session with a CA of 13.7 minutes and a median of 6 log-ins over 12 months. Kramer et al [34] observed higher engagement levels, with participants logging in to PACO, a web-based eHealth service in which 2 ECAs engage in dialogue with an older adult, an average of 39.97 times and spending most of their time on food diaries (85.45%). In Kramer et al [34], engagement tended to decrease over time. In Lee et al [33], engagement was classified as active or passive based on whether the data was entered before or after the daily reminder from the chatbot at 8 PM. Only a small percentage (22.5%) of the data was categorized as active engagement, and 71.8% was classified as passive engagement.

#### User Experience

User experience is well documented (7/11, 63%) and was generally positive, but highlighted areas for improvement. Participants appreciated the likability [33,36,41], ease of use [33,36,41], and tailored recommendations provided by CAs [36,41]. Explanations accompanying recommendations improved satisfaction and trust [36]. However, Kramer et al [34] reported lower ratings for enjoyment and perceived usefulness, with 94% of participants unwilling to pay for the service. Maher et al [38] noted that participants with limited smartphone skills relied on family members for assistance. Other challenges included patronizing tones [33,34] or unnatural conversation [33,35], simplistic content [22] or need for more information [33,35], and perceptions of CAs as unrealistic [33,34]

#### Feasibility and Usability

Finally, assessments of feasibility and usability (2/11, 18%) were encouraging. Maher et al [38] achieved a recruitment target of 30 participants within 6 weeks and a 75% retention rate over 12 weeks, with 70% of participants meeting engagement targets. Kramer et al [34] reported usability scores above the midpoint, with esthetics significantly correlating with usability (*r*=0.44; *P*=.01). Lee et al [33] found a high usability according to the Chatbot Usability Questionnaire. Recruitment was completed in 4 days, and the retention rate at the end of the intervention was 95.2%, with daily participation rates ranging from 83.3% to 100%.

### Additional Results

Although not all the studies included reported supplementary results, some notable observations were made and are worth noting (Table S4 in Multimedia Appendix 2 [22,32-41]). Bickmore et al [41] found that weight change over 2 months was not significantly different between the intervention and control groups. Kramer et al [34] also found no significant changes in quality of life, autonomy, or competence over time, but identified several correlations, including quality of life, autonomy, relatedness, and number of chat messages that were associated with loneliness (although they did not predict loneliness), while esthetics correlated with usability, and enjoyment correlated with perceived usefulness. In addition, perceived usefulness and enjoyment were associated with greater total use time. In contrast, Maher et al [38] reported a modest but statistically significant total weight loss of 1.3 kg (95% CI −2.5 to −0.7; *P*=.01) from baseline to week 12 and a reduction in waist circumference of 2.1 cm (95% CI −3.5 to −0.7; *P*=.003) over the same period. However, no changes in systolic or diastolic blood pressure were observed. The remaining studies (8/11, 7%) [22,32,33,35-37,39,40] did not report additional results beyond the primary and secondary outcomes discussed previously.

Included studies (4/11, 36%) have proposed several recommendations to enhance user experience and engagement with CAs (Table S5 in Multimedia Appendix 2 [22,32-41]). Among those, optimizing message timing to nonworking hours and allowing free-text questions [22], along with a broader range of response options [36], would enable more personalized interactions [22,35]. Integrating CAs with popular platforms like Instagram and WhatsApp, alongside a standalone app, would improve accessibility [22]. Diversifying formats (eg, voice and video) and tailoring content to different age groups and cultural contexts, with translations and adapted to local dietary recommendations, are vital for inclusivity [22]. Flexible conversation lengths [22,32], clear recommendations, short-term health goals [35], and a more natural voice would make interactions more user-friendly and engaging, empowering individuals to manage their health effectively [35].

### Critical Appraisal of the Included Studies

The quality of the included studies was assessed using MMAT [13].

In our review, the 2 qualitative studies [22,35] and the quantitative descriptive study were of very good quality (5 stars) [38], the majority of the quantitative randomized controlled trials (RCTs; 5/6) [34,36,37,40,41] were of average quality (3 stars) to very good quality (5 stars) [39], although 1 quantitative RCT [36] was scored with 1 star because the outcome data were incomplete and the study did not provide sufficient information to determine whether the outcome assessors were blinded to the intervention. In contrast, the quality of mixed method studies varies. One study [32] was awarded 2 stars for quality because participants were not representative of the target population, and outcome data were incomplete. While the other study [33] has been awarded 4 stars (Table S6 in Multimedia Appendix 2 [22,32-41]).

## Discussion

### Principal Findings

This mixed systematic review examined the effectiveness of CAs in improving dietary behaviors. Results indicated improvements in fruit and vegetable intake, adherence to the Mediterranean diet, and nutritional knowledge. Some studies also reported benefits related to PA and stress management. However, in the case of stress, effects were observed in multibehavior interventions not specifically focused on nutrition and thus may reflect broader outcomes rather than direct impacts on dietary behavior. Users generally reported positive experiences, particularly regarding goal setting and tailored feedback, which appeared to enhance motivation to adopt healthier eating habits. Nevertheless, challenges such as limited long-term engagement, inconsistent impacts on social support, and heterogeneity in study design highlight the need for further refinement of CA-based interventions.

It is also important to emphasize that while some improvements did not always reach statistical significance, they may still represent clinically meaningful effects, especially in the context of dietary behavior change, where even modest shifts can contribute to long-term health benefits. For example, even minor enhancements, such as slight reductions in weight and waist circumference, have the potential to yield substantial health benefits on a population level.

### Comparison With Prior Work

Recent evidence reinforces the value of explicitly integrating behavior change theories into the design of CA interventions. A recent scoping review [45] shows that theory-based designs enhance CAs’ effectiveness in promoting healthy behaviors and improve reproducibility, evaluation, and synthesis of findings.

Our review confirms that many CAs were developed using theoretical models, contributing to the observed impacts. Behavior-change frameworks like the TTM, SDT, social cognitive theory, and fuzzy-trace theory provided structure and alignment with key behavioral determinants, including motivation, self-efficacy, and decision-making processes [46-49]. For instance, interventions grounded in the TTM have consistently shown significant improvements in dietary behaviors, including increased fruit and vegetable intake and reduced fat consumption, across diverse populations [50,51]. Recent findings confirm the utility of the TTM in supporting transitions between stages of change and preventing chronic diseases by strengthening self-efficacy and tailoring content to users’ readiness to change [52]. In digital health, the TTM is commonly used to guide behavior change programs by offering stage-matched content, self-assessments, and personalized feedback, with real-time monitoring and adaptive messaging to sustain self-efficacy and support long-term adherence to healthy eating patterns [52,53]. Similarly, studies leveraging SDT have shown that fostering autonomy, competence, and relatedness significantly enhances intrinsic motivation to adopt and maintain healthy eating habits [49,54-56]. In digital health interventions, SDT is used to design features that support user autonomy, competence, and relatedness [57]. Recent digital interventions embedding SDT principles report higher user engagement, stronger intentions to change, and greater satisfaction [58]. Tailoring content to users’ motivational profiles further supports sustained behavior change and improved adherence, particularly in dietary and PA interventions [57,59].

Interventions based on fuzzy-trace theory improve decision-making and long-term adherence to healthy behaviors by simplifying information into meaningful gist representations [60,61]. In digital health, this approach aligns content with users’ core values while reducing cognitive load, improving adherence to health recommendations [62].

Similarly, in digital contexts, social cognitive theory provides a robust framework emphasizing self-efficacy, observational learning, and reinforcement. Interventions grounded in this theory have improved PA and treatment adherence in primary care and mHealth apps [63].

Beyond informing content, these theories strengthen intervention design by providing structured and replicable frameworks for understanding and influencing behavior change. They help identify key behavioral determinants, guide intervention functions, and enable evidence-based techniques tailored to users’ cognitive, emotional, and motivational states [63,64]. Recent reviews have demonstrated that theory-based digital health interventions outperform those without a theoretical grounding. For CAs, theories also inform interaction strategies, timing, and adaptation mechanisms, which are essential for dynamic, responsive user experiences [64]. These findings underline the foundational role of theoretical models in designing effective CA interventions that address the complexity of behavior change.

Behavioral and psychological techniques were also innovatively applied in CA designs. Motivational interviewing, used in 2 studies [32,41], helped CAs elicit “change talk” and address user ambivalence, thereby increasing commitment to dietary goals [65]. Recent evidence suggests that CAs using motivational interviewing principles can enhance users’ willingness to change, particularly when interactions are designed to promote cooperation and reflective thinking [66].

Nudging techniques, as in Pecune et al [36], subtly guided users toward healthier food choices by highlighting preferred options without limiting freedom of choice [67]. In digital health, nudging improves adherence to treatment and dietary choices through adjustments in choice architecture and visual emphasis [68,69]. Similarly, fuzzy-trace theory supports intuitive, gist-based messaging to improve decision-making and comprehension [70].

Persuasion techniques, when applied ethically, strengthen the credibility and emotional resonance of CA interactions and potentially increase their effectiveness [35,36,40,41]. Recent studies show that persuasive dialogue, aligned with users’ emotional and cognitive openness, can influence health behaviors [71]. Theory-based interventions help operationalize such techniques (eg, goal setting, feedback, and self-monitoring) and aligning them with validated mechanisms of action. For instance, social cognitive theory supports reinforcement, modeling, and self-monitoring strategies to improve engagement and outcomes [63].

Several studies integrated empathy [32,35-37] and cultural sensitivity [37,40] to enhance user engagement. Empathy fosters trust, rapport, and promotes positive user experiences, while culturally tailored interventions increase relevance and acceptability among diverse populations [72].

Furthermore, the reviewed studies underscored discrepancies between single-component and multicomponent interventions. Single-component interventions, which focus exclusively on dietary behavior, often exhibit limited effects due to their inability to address the multifactorial nature of behavior change.

In contrast, multicomponent interventions, such as those combining dietary guidance with PA promotion or stress management, tended to yield more significant and sustained outcomes. For instance, Bickmore et al [41] demonstrated that multifaceted interventions produced substantial improvements in fruit and vegetable intake and PA levels. Similarly, interventions targeting multiple lifestyle domains, such as diet, PA, sleep, and stress management, have shown positive outcomes beyond dietary behaviors alone. For example, studies by Dhinagaran et al [22] and Gardiner et al [40] reported that CAs promoting stress-reduction strategies, such as deep breathing and mindfulness, were associated with improved stress management and sleep quality, reinforcing the added value of multicomponent approaches in addressing interconnected health behaviors. These findings align with prior reviews suggesting that comprehensive designs better address the complex interplay of factors influencing behavior change [73]. Such designs are particularly important for addressing co-occurring barriers to change, such as stress and physical inactivity, which often undermine dietary efforts.

A notable strength of CAs is their adaptability to specific populations. For example, Gabby [37,40], developed for African American women, addressed cultural barriers and systemic inequities, fostering trust and relevance in health care interactions. Similarly, Herman and Ellen [34] targeted older adults, providing tailored support for age-related challenges such as accessibility and social isolation. These examples underscore the importance of designing culturally sensitive and demographically tailored CAs. For African American women, cultural relevance, addressing health care distrust, and personalized support for chronic disease prevention are critical. For older adults, interventions must focus on accessibility, cognitive engagement, and reducing isolation. For this population, technology adoption itself can pose a barrier; for example, interventions that are overly complex may discourage participation. Designing intuitive interfaces, including age-appropriate features, and offering support or training could improve adoption and engagement [74,75]. These findings are consistent with studies emphasizing the pivotal role of trust-building, empathy, and personalized interventions in enhancing digital health outcomes [76].

Studies reported improved nutritional knowledge, particularly in understanding food labels and energy balance. For instance, Reyna et al [70] highlighted how intuitive, gist-based messages improve user comprehension, while motivational interviewing and nudging techniques enhance user motivation and adherence. However, challenges remain in achieving consistent improvements in self-efficacy and behavioral intentions, emphasizing the need for interventions that comprehensively address psychosocial determinants of behavior. Integrating real-time feedback and goal-setting mechanisms may enhance these dimensions by providing users with actionable insights and reinforcing positive behaviors. In addition, insights from other digital health research suggest that engagement can be reinforced through mechanisms such as reminders, routine follow-ups, occasional face-to-face contact, or even involving family members in the intervention process [77,78]. These approaches could support long-term adherence.

Future research should prioritize rigorous, long-term studies to evaluate the sustainability and scalability of CA interventions. Expanding inclusion of underrepresented populations, particularly in low-resource settings, is critical for promoting equity and accessibility [79,80]. Attention should also be paid to the modes of delivery, as interventions were deployed through smartphones, computers, or web-based applications, each with distinct usability and accessibility implications [21,81]. Hybrid models combining CA-driven support with human coaching hold promise for addressing engagement and trust challenges [82]. In addition, standardizing outcome measures and leveraging validated theoretical frameworks will be essential for refining CA design and implementation. Ensuring the credibility of content is another critical consideration. CAs should be designed to deliver evidence-based, regularly updated information, ideally developed with input from multidisciplinary teams including clinicians, dietitians, and patients, to guarantee accuracy and personalization [83]. These advancements are crucial for scaling effective and equitable interventions to support global health initiatives and prevent chronic diseases.

### Strengths

This review has several notable strengths. First, to our knowledge, it is the first mixed methods systematic review to specifically examine the effectiveness of CAs in improving dietary behaviors within the general population. Second, by combining quantitative and qualitative evidence, the review provides a comprehensive overview, capturing both the measurable impact of CAs and the user experience. Third, this review systematically documents the theoretical underpinnings, design features, and behavior change techniques embedded in the interventions, offering insights into the mechanisms contributing to their effectiveness. Fourth, rigorous methodological procedures were followed, including duplicate screening, independent quality appraisal, and structured synthesis. These procedures enhance the transparency, reproducibility, and reliability of the findings. Finally, the review identifies knowledge gaps while highlighting promising design strategies, such as theory-based frameworks, personalization, and multicomponent approaches, that could inform the development of more effective, equitable, and scalable CA-based interventions in the future.

### Limitations

This review highlights the potential of CAs to promote dietary behavior change while also revealing key challenges that limit the generalizability and impact of the findings. First, a common issue across the included studies was the focus on specific populations, such as African-American women or individuals with high digital literacy, and the fact that most interventions were conducted exclusively in English. This linguistic and cultural narrowness limits the generalizability of findings and may exacerbate existing health disparities by excluding individuals with lower digital literacy or from non-English–speaking backgrounds.

Many studies relied on participants recruited from online platforms or health-focused communities, introducing selection bias, as these individuals were often already motivated to adopt healthier behaviors.

Second, methodological constraints also impacted the robustness of the findings. Self-reported data were commonly used to measure dietary adherence or behavior changes, which, while convenient, are prone to bias and may overestimate the effectiveness of interventions. Small sample sizes further reduced the reliability of results, and the absence of control groups in certain studies made it challenging to isolate the effects of CA from other factors. Moreover, the short duration of many interventions limited insights into the long-term sustainability of observed changes. While some studies demonstrated initial improvements in dietary behaviors or motivation, follow-up data often failed to confirm whether these benefits persisted.

In addition, differences across study designs (eg, RCTs vs pre-post or qualitative studies), target populations (adolescents, adults, or older adults), and types of CAs (ECA vs RBCA) may have contributed to the heterogeneity of findings. Although our review was not structured by these subgroups, recognizing such variability is important for interpreting results and underscores the need for future reviews or meta-analyses to systematically examine subgroup effects.

Third, very few studies thoroughly evaluated engagement with CAs, usability, or feasibility, limiting the ability to assess their true impact in real-world settings. This lack of process-oriented evaluation makes it difficult to understand how users interact with the CA, what features drive sustained use, and whether the interventions are scalable or adaptable to diverse health system contexts.

Fourth, 3 studies incorporated established dietary guidelines into their interventions. However, adherence outcomes were not always quantitatively measured. Only 1 study [29] explicitly reported measurable improvements in their Mediterranean diet adherence scores, thereby leaving the impact of these guidelines uncertain.

Finally, an additional limitation of this review is the exclusion of gray literature, including dissertations, theses, and reports from relevant organizations. Due to time constraints and limited human resources, it was not possible to conduct a systematic search of these sources as originally planned. As a result, it is possible that relevant studies were missing. This could limit the comprehensiveness of the review and underrepresent the diversity of CA-based interventions being explored in practice, especially in nonacademic or community settings.

These limitations underscore the need for more comprehensive testing and validation of CA-based interventions. Addressing these limitations through more inclusive participant recruitment, enhanced technological adaptability, rigorous methodologies, and long-term evaluations will be essential for maximizing the potential of CA to promote sustainable dietary behavior change.

### Future Directions

To build on the potential of CA while addressing the challenges identified in this review, future research must adopt a multidimensional approach. First, studies should prioritize the design and implementation of larger, longer-term interventions with robust follow-up periods to assess the durability of behavior changes over time. Understanding the characteristics and needs of diverse populations is critical to ensure interventions are culturally sensitive, linguistically accessible, and equitable. Particular attention should be given to underserved and marginalized groups to expand the applicability of findings and maximize public health impact.

Future research should prioritize the development of culturally sensitive and linguistically diverse CAs to ensure greater inclusivity and applicability. Designing interventions in multiple languages and tailoring them to different cultural contexts is essential for reducing inequities and extending the benefits of CA-based dietary interventions to underserved populations in digital health research.

Developing standardized methodologies is another essential step. The adoption of validated outcome measures will enable a more comprehensive assessment of intervention efficacy. These efforts align with the World Health Organization’s Global Strategy for Digital Health 2020‐2025 [84], which advocates for rigorous evaluation frameworks to guide digital health innovations.

Advancements in CA design and functionality will also be central to improving their effectiveness. Enhancing natural language processing capabilities, incorporating advanced personalization algorithms, and designing more intuitive user interfaces will make these technologies more engaging and accessible. Future research should explore how to seamlessly integrate CA into existing clinical workflows through pilot programs and cost-effectiveness analyses. By demonstrating real-world applicability, these initiatives can support the scalability and sustainability of CA-based interventions within health systems.

Equitable access must remain a guiding principle in the evolution of CAs. Bridging the digital divide by addressing barriers to technology adoption, such as literacy gaps, affordability, and cultural relevance, will be critical. Leveraging partnerships with community organizations and public health agencies could further ensure that these interventions reach the populations most in need.

By addressing current challenges and embracing opportunities for innovation, future research can enhance the role of CA in promoting dietary behavior change, preventing chronic diseases, and supporting the integration of these tools into mainstream health care. Such advancements will unlock the full potential of CA to drive meaningful and sustainable health outcomes on a global scale.

### Conclusions

This review underscores the promising yet mixed potential of CA in promoting dietary behavior change. While notable studies demonstrate improvements in dietary intake, adherence to healthy eating patterns, and nutritional knowledge, others report limited or non-significant differences, highlighting variability in study designs, content delivery, CA types, and individual motivation. Positive user experiences across most interventions suggest the feasibility of using CA in health promotion. However, concerns about user engagement, satisfaction, and perceived usefulness reveal critical areas for refinement.

Future research should address existing limitations, including small sample sizes, short study durations, and methodological inconsistencies, while exploring ways to enhance the relevance and inclusivity of CA for diverse populations. Focus should be placed on addressing the barriers faced by underserved communities, including limited digital literacy, language constraints, and socioeconomic challenges, to ensure equitable access to these interventions. Integrating CA into broader health care systems, improving their design through advanced artificial intelligence–driven personalization, and evaluating their long-term public health impact will be essential for maximizing their effectiveness. By prioritizing these advancements, CA could play a transformative role in nutrition and chronic disease prevention, providing scalable and accessible tools for global health promotion.

## Supplementary material

10.2196/78220 Multimedia Appendix 1 Search strategy.

10.2196/78220 Multimedia Appendix 2 Results.

10.2196/78220 Checklist 1 PRISMA checklist.

## Abbreviations

AHA: American Heart Association
CA: conversational agent
ECA: embodied conversational agent
IQR: Interquartile range
MMAT: Mixed Methods Appraisal Tool
PA: physical activity
PICOS: Population, Intervention, Comparison, Outcomes, Study Design
PRISMA: Preferred Reporting Items for Systematic Reviews
RBCA: rule-based conversational agent
RCT: randomized controlled trial
SDT: self-determination theory
TTM: transtheoretical model
