# Supplementary material for: Interactive Conversational Agents to Improve Dietary Behaviors for Health Promotion: Mixed Systematic Review
Source: J Med Internet Res. 2025 Nov 28;27:e78220. doi: 10.2196/78220 (PMC12670327; doi:10.2196/78220)
Supplement: Multimedia Appendix 1 [file jmir-v27-e78220-s001.docx]

**Supplementary materials – Database search strategy**

Medline (OVID)

Date of the search: 2023-10-16 (Update: 2024-12-17)

Database limit: english or french and last 10 years (2013-2023)

| # | Search strategy | Results |
| --- | --- | --- |
| 1 | (chat bot? or chatterbot? or chatbot? or medbot? or chatter bot? or smart bot? or smartbot? or foodbot? or food chatbot or dietbot? or diet bot or voice recognition software or speech recognition software).ti,ab,kw | 1192 |
| 2 | (Conversational adj2 (host or coach or avatar or advisor or assistant or interface or agent? or system or computer or humanoid or character or bot? or AI)).ti,ab | 493 |
| 3 | ((virtual or intelligent or chat or computer or AI or artificial intelligence or relational or embodied or dialogue or natural language processing) adj2 (agent? or assistant? or coach? or system? or companion).ti,ab | 19 990 |
| 4 | Natural language processing/ | 6236 |
| 5 | Artificial Intelligence/ | 40642 |
| 6 | 1 or 2 or 3 or or 4 or 5 | 64 262 |
| 7 | (eating behavio?r or feeding behavio?r or eating habit* or diet* behavio?r or diet* habit* or health* eating or health* diet* or diet* or nutrition* or eating).ab,ti. | 997 196 |
| 8 | Feeding Behavior/ | 93006 |
| 9 | Diet, Healthy/ | 6901 |
| 10 | 7 or 8 or 9 | 1 038 868 |
| 11 | 6 and 10 | 669 |
| 12 | limit 11 to ((english or french) and last 10 years) | 456 |

Web of Science (Clarivate)

Date of the search: 2023-10-16 (Update: 2024-12-17)

Database limit: english or french and last 10 years (2013-2023)

| # | Search strategy | Results |
| --- | --- | --- |
| 1 | TS=(chat bot* or chatterbot* or chatbot* or medbot* or “chatter bot*” or “smart bot*” or smartbot* or foodbot* or “food chatbot” or dietbot* or “diet bot” or “voice recognition software” or “speech recognition software”) | 8827 |
| 2 | TS=(Conversational) NEAR/2 (host or coach or avatar or advisor or assistant or interface or agent* or system or computer or humanoid or character or bot* or AI) | 4981 |
| 3 | TS=(virtual or intelligent or chat or computer or AI or “artificial intelligence” or relational or embodied or dialogue OR “natural language processing”) NEAR/2 (agent* or assistant* or coach* or system* or companion) | 194 933 |
| 4 | 1 or 2 or 3 | 204 495 |
| 5 | TS=(“eating behavio?r” or “feeding behavio?r” or “eating habit*” or “diet* behavio?r” or “diet* habit*” or “health* eating” or “health* diet*” or diet* or nutrition* or eating) | 1 577 636 |
| 6 | 4 and 5 | 1008 |
| 7 | limit 6 to ((english or french) and last 10 years) | 656 |

Embase (Embase.com)

Date of the search: 2023-10-16 (Update: 2024-12-17)

Database limit: english or french and last 10 years (2013-2023)

| # | Search strategy | Results |
| --- | --- | --- |
| 1 | ("chat bot$" OR chatterbot$ OR chatbot$ OR medbot$ OR "chatter bot$" OR "smart bot$" OR smartbot$ OR foodbot$ OR “food chatbot” OR dietbot$ OR “diet bot” OR “voice recognition software” OR “speech recognition software”):ti,ab,kw | 1383 |
| 2 | (Conversational NEAR/2 (host OR coach OR avatar OR advisor OR assistant OR interface OR avatar OR agent$ OR system OR computer OR humanoid OR character OR bot$ OR AI)):ti,ab | 469 |
| 3 | ((virtual OR intelligent OR chat OR computer OR AI OR "artificial intelligence" OR relational OR embodied OR dialogue OR “natural processing language”) NEAR/2 agent$ OR assistant$ OR coach$ OR system$ OR companion)):ti,ab | 25 330 |
| 4 | 'natural language processing'/exp/mj OR 'chatbot'/exp OR 'artificial intelligence'/exp/mj | 46 948 |
| 5 | #1 or #2 or #3 or #4 | 70 319 |
| 6 | (“eating behavio?r” or “feeding behavio?r” or “eating habit$” or “diet$ behavio?r” or “diet$ habit$” or “health$ eating” or “health$ diet$” or diet$ or nutrition$ or eating):ti,ab | 901 016 |
| 7 | 'feeding behavior'/exp/mj OR 'healthy eating'/exp OR 'healthy diet'/exp/mj | 80 338 |
| 8 | #6 OR #7 | 949 461 |
| 9 | #5 AND #8 | 577 |
| 10 | limit 10 to ((english or french) and last 10 years) | 419 |

PsycINFO (OVID)

Date of the search: 2023-10-16 (Update: 2024-12-17)

Database limit: english or french and last 10 years (2013-2023)

| # | Search strategy | Results |
| --- | --- | --- |
| 1 | (chat bot? or chatterbot? or chatbot? or medbot? or chatter bot? or smart bot? or smartbot? or foodbot? or food chatbot or dietbot? or diet bot or voice recognition software or speech recognition software).ti,ab. | 482 |
| 2 | (Conversational adj2 (host or coach or avatar or advisor or assistant or interface or agent? or system or computer or humanoid or character or bot? or AI)).ti,ab | 480 |
| 3 | (virtual or intelligent or chat or computer or AI or artificial intelligence or relational or embodied or dialogue or natural language processing) adj2 (agent? or assistant? or coach? or system? or companion).ti,ab | 8275 |
| 4 | natural language processing/ or chatbots/ or conversational agents/ or human robot interaction/ or intelligent personal agents/ | 2611 |
| 5 | artificial intelligence/ or intelligent agents/ | 12 629 |
| 6 | 1 or 2 or 3 or or 4 or 5 | 21 |
| 7 | (eating behavio?r or feeding behavio?r or eating habit* or diet* behavio?r or diet* habit* or health* eating or health* diet* or diet* or nutrition* or eating).ab,ti. | 118 887 |
| 8 | eating behavior/ or healthy eating/ | 17 236 |
| 9 | 7 or 8 | 122 165 |
| 11 | 6 and 9 | 117 |
| 12 | limit 11 to ((english or french) and last 10 years) | 75 |

CINAHL

Date of the search: 2023-10-17(Update: 2024-12-17)

Database limit: english or french and last 10 years (2013-2023)

| # | Search strategy | Results |
| --- | --- | --- |
| 1 | TI ("chat bot?" OR chatterbot? OR chatbot? OR medbot? OR "chatter bot?" OR smart bot? OR smartbot? OR foodbot? OR “food chatbot” OR dietbot? OR “diet bot” OR “voice recognition software” OR “speech recognition software”) OR AB ("chat bot?" OR chatterbot? OR chatbot? OR medbot? OR "chatter bot?" OR smart bot? OR smartbot? OR foodbot? OR “food chatbot” OR dietbot? OR “diet bot” OR “voice recognition software” OR “speech recognition software”) | 598 |
| 2 | TI (Conversational N2 (host OR coach OR avatar OR advisor OR assistant OR interface OR avatar OR agent? OR system OR computer OR humanoid OR character OR bot? OR AI) OR AB (Conversational N2 (host OR coach OR avatar OR advisor OR assistant OR interface OR avatar OR agent? OR system OR computer OR humanoid OR character OR bot? OR AI)) | 228 |
| 3 | TI ((virtual OR intelligent OR chat OR computer OR AI OR "artificial intelligence" OR relational OR embodied OR dialogue OR “natural language processing”) N2 (agent? OR assistant? OR coach? OR system? OR companion) OR AB ((virtual OR intelligent OR chat OR computer OR AI OR "artificial intelligence" OR relational OR embodied) N2 (agent? OR assistant? OR coach? OR system? OR companion) | 6141 |
| 4 | (MM "Natural Language Processing") | 1732 |
| 5 | (MM "Artificial Intelligence") | 6150 |
| 6 | S1 OR S2 OR S3 OR S4 OR S5 | 13 621 |
| 7 | TI(“eating behavio?r” or “feeding behavio?r” or “eating habit?” or diet? behavio?r or “diet? Habit?” or “health? eating” or “health? diet?” or diet? or nutrition? or eating) OR AB (“eating behavio?r” or “feeding behavio?r” or “eating habit?” or diet? behavio?r or “diet? Habit?” or “health? eating” or “health? diet?” or diet? or nutrition? or eating) | 214 023 |
| 8 | (MH "Eating Behavior") | 21 209 |
| 9 | S7 OR S8 | 221 642 |
| 10 | (S7 OR S8) AND (S6 AND S9) | 144 |
| 11 | limit 11 to ((english or french) and last 10 years) | 105 |
