# Supplementary material for: Interactive Conversational Agents to Improve Dietary Behaviors for Health Promotion: Mixed Systematic Review
Source: J Med Internet Res. 2025 Nov 28;27:e78220. doi: 10.2196/78220 (PMC12670327; doi:10.2196/78220)
Supplement: Multimedia Appendix 2 [file jmir-v27-e78220-s002.docx]

**Table S1.** Characteristics of the included studies

| **Studies**  **Year**  **Country** | **Study design** | **Aim and intervention** | **Duration** | **Participant characteristics** | **Name** | **Type of conversational agent** | **Specification** | **Theoretical model/**  **Techniques** |
| --- | --- | --- | --- | --- | --- | --- | --- | --- |
| **Bickmore**  **2013 [1]**  **USA** | RCT | **Aim:** To promote FV consumption and PA through simulated conversations.  **Intervention**  Daily contact with the ECA and comparison between 4 groups:  1) **IG 1:** PA group (30 min/day of moderate to intense PA).  2) **IG 2:** FV group (4.5 cups/day of FV).  3) **IG 3:** FV+PA group.  4) **CG:** No intervention. | 2 months | **N=**122.  **Age (Mean):** 33.0 years (SD=12.6).  **Sex/Gender:**  Male: 39.3%.  Female: 60.7%.  **Ethnicity:**  Asian: 32.7%.  Black: 8.0%.  White: 51.6%.  Am. Indian: 0.8%.  **Overweight or obesity status:**  BMI (kg/m^2^): 18.8 to 46.4 (mean 27.8). | Karen | ECA | Text (Chat)  Software  Female avatar | - Transtheoretical model - Social cognitive theory - Motivational interviewing |
| **Brust-Renck**  **2017 [2]**  **USA** | RCT | **Aim:** To investigate the role of active learning using a web-based ITS to create a scalable and cost-effective way to engage many people in dialogue about obesity simultaneously.  **Intervention**  An online tutorial focused on preventing obesity in women by improving understanding of basic nutrition and exercise principles and promoting healthy behaviors related to diet and PA.   - **IG:** Content tutorial: lessons on energy balance, nutrition labels, fast food, advertising, basic nutrition, and exercise fundamentals. - **CG:** Content tutorials: genetic risk and breast cancer risk. | 90 min | **N=**220.  **Age (Mean):** 19.21 years (SD=1.73).  **Sex/Gender:**  Female: 100%.  **Ethnicity:**  Caucasian: 61.5%.  African American: 10%.  Hispanic: 9.1%.  Asian: 21.8%.  Mixed/Other ethnic groups: 6.7%. | GistFit | ECA | Text (Chat)  Voice  Figures and images  Web platform  Female avatar (3 different ethnicities) | - Fuzzy-Trace Theory - Social Cognitive Theory |
| **Dhinagaran**  **2022 [3]**  **Singapore** | Qualitative study | **Aim:** To explore the acceptance, satisfaction, and critical appraisal of a CA that promotes healthy behaviors in the general population.  **Intervention**   - Conversations with users initiated by the CA four times a week (16 sessions total). - Targeted lifestyle behaviors: diet, exercise, sleep and stress. | 1 month | **N=**20.  **Age (Mean):** 33 years (SD=9.23).  **Sex/Gender:**  Female: 60%.  **Ethnicity:**  Caucasian: 5%.  Chinese: 75%.  Indian: 15%.  Malay: 5%. | Precilla | RBCA | Text (Chat)  Images  Video links  Facebook messenger App  Female avatar | NS |
| **Gardiner**  **2017 [4]**  **USA** | RCT | **Aim:** To evaluate the feasibility of using an ECA to teach urban women lifestyle modifications (stress management, healthy eating, and PA).  **Intervention**  **IG:**   - Use of an ECA to teach lifestyle modifications to urban women, allowing free and flexible interactions in frequency and duration.   **CG:**   - Information sheets and audio resources (CD/MP3) providing the same meditations, exercises, and content as the ECA. - Daily listening to recordings and unlimited reading of the sheets. | 1 month | **N**=61.  **Age** (Mean): 35 years (SD= 8.4).  **Sex/Gender:**  Female: 100%.  **Ethnicity:**  White: 51%.  Black: 26%.  Asian/Pacific Islander: 2%.  Other: 21%. | Gabby | ECA | Text (Chat)  Voice  Web platform  Female avatars (African American) | - Transtheoretical model |
| **Gardiner**  **2021 [5]**  **USA** | RCT | **Aim:** To study the impact of the ECA on a subset of women who reported at least one of the ten preconception health risks in the nutrition domain.  **Intervention**   - Health risk assessment and tailored dialogue on preconception health, focusing on nutrition and supplement risks, provided by an online ECA.A customizable list of health topics generated by the ECA for discussion. - 2 groups:   **IG:**   - Log in at least 1x/2 weeks. - Topics addressed with the ECA: Food choices, supplements needed for preconception care, supplements, and chemicals for? safe consumption.   **CG:**   - Participants received a letter listing the health risks reported on the risk assessment. - Participants were encouraged to discuss their risks with a health professional. | 12 months | **N=**480.  **Age** (Mean): 27 years (SD=3.91).  **Sex/Gender:**  Female:100%.  **Ethnicity:**  Hispanic: 9.6%.  **Obesity status:**  BMI (kg/m^2^) > 30: 82.9% (SD=NS). | Gabby | ECA | Text (Chat)  Voice  Web platform  Female avatars (African American) | - Transtheoretical model - Motivational interviewing - Shared decision making |
| **Kramer**  **2022 [6]**  **Netherland** | RCT | **Aim:** To (1) identify whether ECAs could persuade community-dwelling older adults to change their dietary behavior and whether ECA uses? could decrease loneliness, (2) test the pathways to these effects, and (3) understand factors influencing the use of ECAs.  **Intervention**   - eHealth service where two ECAs motivate users to improve eating habits and reduce loneliness. - 5 modules, including nutrition, each using a different behavior change techniques. - Topics covered in the nutritional module: intake of FV, meat, and liquids and healthy recipes.   **IG (cohort 1):**   - Dialogue with the two ECAs.   **CG (cohort 2):**   - Waitlist group began using the ECAs after a 4-week waiting period. | 2 months | **N=**32.  **Age** (Mean): 73 years (SD=5.33).  **Sex/Gender:**  Women: 56%.  **Ethnicity:** NS. | Herman and Ellen | ECA | Text (Chat)  Web platform  Avatar (male and female) | - Self-Determination Theory - Transtheoretical model - Persuasion |
| **Lee**  **2024 [7]**  **Republic of Korea** | Pre-post study | **Aim:** To evaluate the usability and effectiveness of a behavior change technique (BCT)-based  chatbot developed to promote healthier beverage consumption among adolescents.  **Intervention**   - The ECA analyzes beverage (carbonated and/or energy drinks) consumption data and provides feedback based on the users’ beverage intake. - Participants must enter their fluid consumption data by 8 pm each day. | 2 weeks | N=42.  **Age** (Mean): 15.0 years (SD=0.7)  **Sex/Gender:**  Women: 69%.  Men: 31%.  **Ethnicity:** NS. | R-Ma Bot | RBCA | Text (Chat)  Social media | - Transtheoretical model - Feedback and rewards |
| **Maher**  **2020 [8]**  **Australia** | Pre-post study | **Aim:** To test the feasibility and preliminary efficacy of PA and Mediterranean-style dietary intervention (MedLiPal) delivered via AI virtual health coach.  **Intervention**   - ECA-guided sessions with personalized introductions, weekly check-ins, goal setting, and Q&A support. - Participants used a tracker to monitor daily steps, a website with educational materials and recipes, and a printed diet and activity log sheet. | 3 months | **N=**31.  **Age** (Mean): 56.2 years (SD=8.0).  **Sex/Gender:**  Women: 67%.  Men: 33%.  **Ethnicity:** NS.  **Overweight or obesity status:**  Overweight: 32%.  Obese: 42%. | Paola | ECA | Text (Chat)  Slack application  Avatar (female) | - Transtheoretical model |
| **Pecune**  **2022 [9]**  **UK** | RCT | **Aim:** To investigate how the conversational skills of a recipe recommender system and the nudges it relies on would influence users’ perception and their intention to cook.  **Intervention**   - The system recommends recipes matching users’ needs while building rapport with them. - The system proposes alternatives and justifies its recipe recommendation by explaining their trade-offs. - System evaluation through two experim**e**nts:   **Experiment 1:** To study the impact of conversational skills’ system and interaction mode on its persuasiveness and users’ experience.  **Experiment 2:** To evaluate whether recipe comparisons and recommendation explanations significantly impact users’ perception of a healthy recipe recommender system. | One time exposition | **N=**NS.  **Age (Mean):** NS.  **Sex/Gender:**  Experience 1: Men: 67 %.  Experience 2: NS.  **Ethnicity:** NS. | Cora | RBCA | Text (Chat)  Buttons and drop-down lists  Pre-defined answers to select  Unclear  Avatar (female) | - Nudging techniques |
| **Saravanan**  **2022 [10]**  **Netherland** | Experimental study (Mixed Methods) | **Aim:** a) To design and evaluate the conversational memory for personalized behavior change support conversations on healthy nutrition via memory-based motivational rephrasing; b) To explore how far motivational rephrasing affects user’s perception of the CA.  **Intervention**  Three conversation sessions with the CA via Zoom:   - Sessions focused on changing eating and exercise habits to improve a person's health. - **Session 1:** To learn about the user and helps them set personal goals and milestones for the following sessions. - **Sessions 2** **and 3:** Goals and milestones are reviewed. The CA refers to the motivational memories from the previous session. - Three conditions are created to evaluate the effects of memory references and ran the experiment as a between-subjects design:  1. Condition I: No references to previous events. 2. Condition II: References to only one event. 3. Condition III: Multiple references to different events. | 3 days | **N=**79.  **Age (Mean):** 31.5 years (SD= NS).  **Sex/Gender:**  Female: 47.4%.  Male: 55.3%.  Nonbinary: 1.3%.  **Ethnicity:** NS. | Furhat | RBCA | Text (Chat)  Web platform  Head of robot (no avatar) | - Motivational interviewing |
| **Smriti**  **2022 [11]**  **USA** | Qualitative study | **Aim:** To develop a CA to improve healthy eating in parents who serve as a proxy for health behavior change in their children.  **Intervention**   - Two user test sessions of the CA prototype via Zoom with parents of young children (20 min/session) to understand the perceived acceptability and usefulness of the prototype. - Session topics: identifying dietary barriers, reflecting on behaviors, setting short- and long-term goals, and assessing progress in an optional follow-up session. | 2 weeks | **N=**24.  **Age (Mean):** 41.08 years (SD=10.53).  **Sex/Gender:**  Female: 63%.  Male: 37%.  **Ethnicity:** NS.  **Overweight or obesity status:**  BMI (kg/m^2^): 31.13 (SD=5.76). | MICA | ECA | Voice  Web platform  Prototype (no avatar) | - Transtheoretical model |

CA: Conversational agent.

IG: Intervention group.

CG: Control group.

PA: Physical activity.

FV: Fruit and vegetables.

NS: Not specified.

BMI: Body Mass Index.

ECA: Embodied Conversational agent.

RBCA: Rule based conversational agent.

MICA: Motivational Interviewing Conversational Agent.

ITS: Intelligent Tutoring System.

NIH: National Institutes of Health.

NCI: National Cancer Institute.

AI: Artificial intelligence.

SD: Standard deviation.

**Table S2.** Primary Outcomes: Dietary behaviors

| **Studies** | **Consumption/intake**  **(Fruit and vegetables/Proteins/Whole grains/Others)** | **Eating behaviors** | **Behavioral intentions** | **Self-efficacy** | **Adherence to dietary recommendations** |
| --- | --- | --- | --- | --- | --- |
| **Bickmore**  **2013 [1]** | **Fruit and vegetables:**   - Significantly higher daily fruit and vegetable consumption observed in the FV group compared to the CG. - Higher fruit and vegetable consumption reported in the FV+PA group compared to the CG, although without statistical significance. | − | − | − | National Institutes of Health (NIH) and National Cancer Institutes (NCI) recommendations |
| **Brust-Renck**  **2017 [2]** | **Fruit and vegetables/Proteins/Whole grains/Others:**   - No significant difference in healthy nutritional behavior (weekly consumption of FV, fish, whole grains, sugar and sodium) was observed between the IG and CG | − | - Behavioral intentions are a predictor of behavior. - Behavioral intentions correlate with adopting a healthy diet (weekly consumption of FV, fish, whole grains, sugar and sodium). - Declared intentions regarding nutrition and overall behavior are linked to adherence to and comprehension of basic principles. | − | American Heart Association (AHA) recommendations |
| **Dhinagaran**  **2022 [3]** | Minimal changes in proteins, fat and fiber consumption. | − | Lack of intention on the part of the participants for a change in their lifestyle. | − | − |
| **Gardiner**  **2017 [4]** | **Fruit and vegetables:**   - No statistically significant increase in vegetable servings observed in either group - Significantly greater increase in fruit servings among CA users compared to information sheet users.¸   **Proteins:**   - No significant difference in the consumption of red meat, and fish between the two groups.   **Whole grains:**   - No significant difference between the two groups.   **Others:**   - Reduction of weekly caffeine and snack consumptions after 1 month in IG compared to the CG but statistically insignificant. - No significant difference in breakfast consumption between groups. | − | − | No significant difference in self-confidence to eat healthy between groups. | − |
| **Gardiner**  **2021 [5]** | − | − | − | − | − |
| **Kramer**  **2022 [6]** | **Fruit and vegetables:**   - CA were not able to persuade users to change their fruit, vegetable intake.   **Others:**   - CA were not able to persuade users to change their liquid intake. | − | − | Competence correlates with eating behavior and predicts eating behavior over time. | − |
| **Lee**  **2024 [7]** | - Significant 60% reduction in weekly sugar intake from beverages, decreasing.from 13.1 ±20.1 mg to 7.9 ±12.8 mg. - No reduction observed in sodium or caffeine consumption from carbonated and/or energy drinks. | − | − | − | − |
| **Maher**  **2020 [8]** | **Fruit and vegetables/Proteins/Whole grains**  **(Mediterranean-style diet adherence):**  **Baseline to 6 weeks:**  Significant increase in adherence to a Mediterranean-style diet.  **6 to 12 weeks:**   - Significant maintenance of Mediterranean-style diet adherence over 12 weeks, with a mean change of 5.7, 95% CI: 4.2 to 7.3, *P*<0.001). | − | − | − | Mediterranean diet style |
| **Pecune**  **2022 [9]** | − | - 47% of users accepted the healthy recipe recommendations across all conditions. - Average health score of accepted recipes was significantly lower than that of users' initially preferred recipes, meaning the accepted recipes were healthier. - Explanations from the CA increased the acceptance rate of healthy recipes, but the difference was not statistically significant. | − | − | − |
| **Saravanan**  **2022 [10]** | - Changes in eating behavior related to calorie restriction or sugar reduction. | - No significant differences between the experimental groups on all milestone and final goal attainment related to calorie restriction or sugar reduction.. - High goal attainment observed, with 86.1% of participants achieving their target (Condition I: 88.5%; Condition II: 88.5%; Condition III: 81.5%). | − | − | − |
| **Smriti**  **2022 [11]** | − | **Influence on children:**   - By improving their eating habits, specifically reducing overeating, sweetened beverage consumption, dining out, fast food intake, stressful eating, and out-of-control eating, parents can better influence their children's eating behaviors, and serve as positive role models.   **Stimulated reflection**:   - CA's ability to encourage personal reflection enables parents to better understand their habits and identify the changes needed to promote healthy eating within the family.   **CA safely working with complex family dynamics:**   - CA should understand and work with complex family dynamics as third-party support. - Parents express concern about the negative influence that other family members may have on their children's eating habits. | − | − | − |

IG: Intervention group.

CG: Control group.

PA: Physical activity.

FV: Fruit and vegetables.

CA: Conversational agent.

NIH: National Institutes of Health.

NCI: National Cancer Institute.

AHA: American Heart Association.

**Table S3.** Secondary Outcomes

| **Studies** | **Nutritional knowledge** | **Other lifestyle behaviors** | **Social support** | **Motivation** | **Engagement** | **User experience** | **Usability /feasibility** |
| --- | --- | --- | --- | --- | --- | --- | --- |
| **Bickmore**  **2013 [1]** | ─ | **Physical activity:**  ***Steps count:***  PA group increases their daily walking faster than CG, but the FV group is slower, and the PA+FV group is in the middle.  ***IPAQ Score:***  No significant differences among conditions, | ─ | ─ | ─ | **Satisfaction with agent/Ease of use/Desire to continue with agent:**   - No significant differences among the three intervention groups.   **In general:**   - Being friendly, personal and, approachable were valued ECA qualities - Showing progress with the steps chart was appreciated - Being non-disciplinary when unmet goals or the system was not accessed was valued. - Achieving goals with the ECA help was noted. - Constant reminders from the ECA were highlighted as the most helpful aspect | ─ |
| **Brust-Renck**  **2017 [2]** | - Improvement in knowledge, understanding (energy balance, food labels, fast food, and advertising) and behaviors related to nutrition. - Self-reported healthy nutrition is related to greater verbatim knowledge. | **Physical activity:**  ***Healthy Exercising Behavior improvement:***  ***PA knowledge:***   - Improvement of knowledge, understanding and behaviors related to PA (e.g. exercise basics). - Self-reported healthy exercising is related to greater verbatim knowledge. | ─ | ─ | Engagement in tutorial dialogue was associated with increased knowledge and transfer of that knowledge by understanding the behaviors preventing obesity. | ─ | ─ |
| **Dhinagaran**  **2022 [3]** | **Nutritional knowledge to prevent diabetes and prediabetes:**  **Positive aspects:**   - Content was detailed and informative. - Content is relevant and helpful in learning new content. - Content reinforces prior knowledge.   **Negative aspects:**   - Irrelevant content was provided for participants who were familiar with healthy living and diabetes prevention or had received similar advice. | **Stress management:**   - Interactions with the ECA had positive effects on stress management. - Advice delivered considered deep breathing, mindfulness and other relaxation techniques.   **Sleep:**   - Positive effects on sleep improvement. | ─ | ─ | ─ | ***Positive aspects:***   - Positive impression. - Likable personality and appropriate tone. - Available, reliable and accurate information. - Pleasant interface and visual aids. - Ease of use and easily digestible content. - Detailed and informative content.   ***Negative aspects:***   - Overall low level of satisfaction. - Simple content. - Long messages. - Content difficult to put into practice (e.g. mindfulness). | ─ |
| **Gardiner**  **2017 [4]** | No significant difference in food knowledge between groups | **Physical activity:**   - IG increased PA more than CG *(P*=0.88). - No significant increase in duration of exercise in both groups for stretching/ strengthening (*P*=0.18) and aerobic exercise (*P*=0.27). - No significant difference in self-confidence to engage in PA between groups. - 52% of participants utilized suggestions from Gabby to increase PA, but not significantly different from CG (49%).   **Stress management:**   - ECA to improve stress management is preferred to patient information sheets. - 70% utilized suggestions from Gabby to manage stress: all of which were greater but not significantly different from CG (66%). - Significant decrease in the number of women who used alcohol for stress management in the IG at one month compared to the CG (35% to 14% for IG, 30% to 31% for CG; | ─ | ─ | - CA users spent a median total of 52 min (IQR=101.4, N=31) interacting with the ECA and logged in 5 times (IQR=7, N=31). - Average time spent per session was 12.1 min. | - 69% of participants utilized suggestions from the CA to improve healthy eating. | ─ |
| **Gardiner**  **2021 [5]** | ─ | ─ | ─ | **Advancing stages of change:**  Behaviors most likely to progress on the stage of change scale and most likely to reach the action or maintenance stage were unhealthy diet, no folic acid supplementation, and no iron supplementation. | - 198 women in the IG interacted with the entire Gabby system at least once, and the median number of logins was 6 (IQR=8) after 12 months. - Median session duration with Gabby was 13.7 min (IQR=8.7 min). - Median average interaction time with Gabby was 73.8 min (IQR=134.6) per woman who used Gabby. - 116 interventions subjects at 6 months, and 105 intervention subjects at 12 months. | ─ | ─ |
| **Kramer**  **2022 [6]** | ─ | − | The CA were not able to persuade users to decrease loneliness | ─ | - Connection frequency: 39.97 times. - Average total time spent: 6 h 30 min. - Time spent per session:11 h 10 min. - Average number of modules used per session: 2.39. - Most of the time was spent on the food diary (85.45%), followed by the recipes (6.36%), goals (4.58%), and stories (3.61%). - Mean of messages sent: 27.78. - Reading the module content, did not truly engage some participants and the content was not helpful for all participants. - Strength of the relationship with the ECAs decreased over time. | ***Positive aspects:***   - Aesthetics, privacy concerns, and perceived control scored above the scale midpoint. - Enjoyment correlated with perceived usefulness. - Perceived usefulness (*r*=0.39, *P*=0.03) and enjoyment (*r*=0.38, *P*=0.03) were linked to usage duration.   ***Negative aspects:***   - Enjoyment and service usefulness were rated below the midpoint. - Perceived usefulness received relatively low ratings. - Most (94%) participants were unwilling to pay for the service. | - Usability is rated above the midpoint of the scale. - Aesthetics correlated significantly with usability |
| **Lee**  **2024 [7]** | - Awareness of nutrition labels increased from 64.3% to 92.9%, and nonreaders decreased from 42.9% to 16.7%. | − | − | − | - Only a small percentage (22.5%) of the data was categorized as active engagement. | ***Positive aspects:***   - Usefulness - Friendliness - Easy   ***Negative aspects:***   - Unnatural conversation - Need more information | **Usability:**   - High usability:   Mean of the Chatbot Usability Questionnaire (CUQ) score for all participants was 74.7 (SD=11.9). Scores ranged from 54.5 to 100.0.   - Recruitment was completed in 2 and 4 days - Retention rate at the end of intervention was 95.2% - Daily participation rates ranged from 83.3% to 100%. |
| **Maher**  **2020 [8]** | − | **Baseline to 6 weeks**:   - Weekly PA increased by approximately one hour, and then by 50 minutes at 12 weeks.   **Baseline to 12 weeks:**   - PA increased by 109.8 minutes (95% CI: 1.9 to 217.7, *P*=0.005). | − | − | − | Participants with minimal smartphone skills relied on their partners or children to use their phones for the study. | **Feasibility based on recruitment, retention, and engagement:**   - Recruitment of 30 participants within 6 weeks. - Retention of 75% at 12 weeks. - Behavioral engagement with the program: assessed via virtual health coach usage¸ data (number of weekly check-ins completed). - Engagement target was set at 70% (e.g., participants would complete at least 8 of 11 weekly sessions with Paola). |
| **Pecune**  **2022 [9]** | ─ | − | ─ | ─ | ─ | - System was rated with higher scores when engaging participants in a social dialogue than a task dialogue. - Cora was perceived as more useful and easier to use when it justified its recommendations with a specific explanation. - For user beliefs, the version of Cora that could explain its recommendations obtained better scores than the one recommended without explanation. - Satisfaction and trust in Cora are significantly higher when it accompanied its recommendations with an explanation. | ─ |
| **Saravanan**  **2022 [10]** | − | − | − | - Significant effect of memory model on participants ‘motivation to engage in behavior change. - Significantly higher motivation increases in participants assigned to condition 3 than participants assigned to condition 1 or condition 2). | - No significant effect of memory model on participants ‘engagement in the interaction between the three conditions. | - No significant differences between the experimental groups in participants ‘perception of the robot. - More than 1/3 of participants would prefer the interaction to be longer while the remainder found the length of the interaction to be appropriate. | − |
| **Smriti**  **2022 [11]** | − | − | − | - MICA helps parents reflect on their eating habits, which motivates them to make changes that benefit themselves and their children. - Participants see MICA as a motivating support that helps them achieve their dietary goals despite the challenges of parenthood. | − | **Interaction with MICA:**   - MICA ask specific questions about food, prompting parents to reflect on their past eating behaviors.   **MICA safety and trust:**   - Participants want MICA to focus on safety before behavior change objectives, providing advice tailored to complex family situations.   **Perception of MICA's support role:**   - MICA is seen as a support system that can help parents maintain consistency in their children's diets, even in separation or shared custody. | − |

IG: Intervention group.

CG: Control group.

PA: Physical activity

FV: Fruit and vegetables.

CA: Conversational Agent.

MICA: Motivational Interviewing Conversational Agent.

IPAQ: International Physical Activity Questionnaire.

CUQ: Chatbot Usability Questionnaire.

IQR: Interquartile range.

SD: Standard deviation.

CI: Confidence interval.

**Table S4.** Other Outcomes reported

| **Studies** | **Other outcomes** |
| --- | --- |
| **Bickmore, 2013 [1]** | **Weight change:** Weight changes for participants over the two months of the study were not statistically different between groups. |
| **Brust-Renck, 2017 [2]** | NA |
| **Dhinagaran, 2022 [3]** | NA |
| **Gardiner, 2017 [4]** | NA |
| **Gardiner, 2021 [5]** | NA |
| **Kramer, 2022 [6]** | - No significant differences over time in quality of life. (Brief older people’s quality of life questionnaire). - No significant differences over time in autonomy, competence, and relatedness. - Quality of life, autonomy and relatedness, and number of chat messages correlated with loneliness (De Jong Gierveld loneliness scale), but did not predict loneliness. - Aesthetics correlated significantly with usability (System usability scale), and enjoyment (Affect scale) correlated with perceived usefulness (Perceived usefulness scale). - Perceived usefulness and enjoyment correlated with use in minutes. |
| **Lee, 2024 [7]** | NA |
| **Maher, 2020 [8]** | **Weight change:**   - Participants lost 1.1 kg from baseline to week 6 and a further 0.2 kg to week 12, resulting in an overall average loss of 1.3 kg (95% CI: –2.5 to –0.7, *P*=0.01).   **Waist circumference change:**   - Waist circumference decreased by 1 cm from baseline to week 6, then another 1 cm to week 12, leading to an overall loss of –2.1cm (95% CI: –3.5 to –0.7, *P*=0.003).   **Blood pressure change:**   - There was no change in blood pressure (diastolic or systolic) at either time point. |
| **Pecune, 2022 [9]** | NA |
| **Saravanan, 2022 [10]** | NA |
| **Smriti, 2022 [11]** | NA |

NA: Not applicable.

CI: Confidence interval.

**Table S5.** Suggestions for improvement

| **Studies** | **Suggestions for improvement** |
| --- | --- |
| **Bickmore, 2013 [1]** | CA needs to empower participants more. |
| **Brust-Renck, 2017 [2]** | NS |
| **Dhinagaran, 2022 [3]** | **Message timing:**   - Send messages before or after work, when people are alone and at home.   **Communication:**   - Expand response options. - Allow free text entry for questions. - Make conversations more humane. - Have more intelligent versions in the future.   **Broadcast platform:**   - Instagram and WhatsApp, more popular with young people. - Possibility of a stand-alone application for Precilla.   **Integration with other platforms:**   - Combine Precilla with an online course for denser content. - Combine Precilla with face-to-face consultations with healthcare professionals.   **Diversifying message types:**   - Include voice or video messages in addition to text. - Improve error messages to clarify the limits of the conversational agent.   **Personalization:**   - Tailor content to specific age groups. - Offer options for more in-depth exploration of certain topics, - Translate content into other languages relevant to Singapore.   **Improve links to websites:**   - Include links to helplines, educational conferences, courses and more detailed information.   **New and additional content:**   - Extend interaction time with new content. - Add information about prediabetes, its signs and symptoms. - Include dietary recommendations specific to the Singapore context. - Clarify the difference between healthy foods and “healthier options |
| **Gardiner, 2017 [4]** | NS |
| **Gardiner, 2021 [5]** | NS |
| **Kramer, 2022 [6]** | NS |
| **Lee, 2024 [7]** | NS |
| **Maher, 2020 [8]** | NS |
| **Pecune, 2022 [9]** | **Recommendations’ limit:**   - Provide no more than two alternatives (recommendations) at a time and clearly explain their differences to increase user engagement.   **Interaction modes:**   - Implement different interaction modes to accommodate users who prefer casual conversation or a more straightforward, efficient recommendation process. - Allow users to type their responses instead of solely relying on menu selections to enhance user experience and accommodate diverse preferences.   **Chat text:**   - Improve Cora's text processing capabilities to better interpret user inputs and reduce frustration during text-based interactions. |
| **Saravanan, 2022 [10]** | NS |
| **Smriti, 2022 [11]** | **Interaction and conversation:**   - Offer options for more natural conversations or a non-human approach to minimize feelings of judgement. - Set up more prolonged interactions that explore the reasons behind participants' responses. - Incorporate relevant personalized questions that help users make informed decisions about their eating habits and their children's. - Offer more direct answers, without positive statements, as some users prefer a more factual approach.   **Goals setting/motivation:**   - Suggest short-term objectives, such as weekly or monthly monitoring periods to keep participants motivated. - **MICA’s voice:** Reduce the robotic quality of MICA's voice to enhance the pleasure and comfort of interaction. |

NS: Not specified.

CA: Conversational agent.

MICA: Motivational Interviewing Conversational Agent.

**Table S6.** Quality assessment of included studies based on the Mixed Methods Appraisal Tool

| **Authors** | **Study design** | **Quantitative RCT** | **Quantitative descriptive** | **Mixed Methods** | **Qualitative** |
| --- | --- | --- | --- | --- | --- |
| **Bickmore, 2013 [1]** | RCT | **** (4 stars) | − | − | − |
| **Brust-Renck, 2017 [2]** | RCT | ***** (5 stars) | − | − | − |
| **Dhinagaran, 2022 [3]** | Qualitative study | − | − | − | ***** (5 stars) |
| **Gardiner, 2017 [4]** | RCT | **** (4 stars) | − | − | − |
| **Gardiner, 2021 [5]** | RCT | **** (4 stars) | − | − | − |
| **Kramer, 2022 [6]** | RCT | *** (3 stars) | − | − | − |
| **Lee, 2024 [7]** | Pre-post study | − | − | **** (4 stars) | − |
| **Maher, 2020 [8]** | Pre-post study | − | ***** (5 stars) | − | − |
| **Pecune, 2022 [9]** | RCT | * (1 star) | − | − | − |
| **Saravanan, 2022 [10]** | Experimental study |  |  | ** (2 stars) |  |
| **Smriti, 2022 [11]** | Qualitative study |  |  |  | ***** (5 stars) |

**References**

1. Bickmore, T.W., D. Schulman, and C. Sidner, *Automated interventions for multiple health behaviors using conversational agents.* Patient Educ Couns, 2013. **92**(2): p. 142-8.

2. Brust-Renck, P.G., et al., *Active engagement in a web-based tutorial to prevent obesity grounded in Fuzzy-Trace Theory predicts higher knowledge and gist comprehension.* Behav Res Methods, 2017. **49**(4): p. 1386-1398.

3. Dhinagaran, D.A. and L.T. Car, *Public perceptions of a healthy lifestyle change conversational agent in Singapore: A qualitative study.* Digit Health, 2022. **8**: p. 20552076221131190.

4. Gardiner, P., et al., *Engaging Women with an Embodied Conversational Agent to Deliver Mindfulness and Lifestyle Recommendations: A Feasibility Randomized Control Trial.* Patient Education and Counseling, 2017. **100**.

5. Gardiner, P., et al., *Using Health Information Technology to Engage African American Women on Nutrition and Supplement Use During the Preconception Period.* Front Endocrinol (Lausanne), 2020. **11**: p. 571705.

6. Kramer, L.L., et al., *Use and Effect of Embodied Conversational Agents for Improving Eating Behavior and Decreasing Loneliness Among Community-Dwelling Older Adults: Randomized Controlled Trial.* JMIR Form Res, 2022. **6**(4): p. e33974.

7. Lee, J., H. Lee, and H. Lee, *Navigating healthier beverage consumption in adolescents using the "R-Ma Bot" chatbot: A usability and evaluation study.* Digit Health, 2024. **10**: p. 20552076241283243.

8. Maher, C.A., et al., *A Physical Activity and Diet Program Delivered by Artificially Intelligent Virtual Health Coach: Proof-of-Concept Study.* JMIR Mhealth Uhealth, 2020. **8**(7): p. e17558.

9. Pecune, F., L. Callebert, and S. Marsella, *Designing Persuasive Food Conversational Recommender Systems With Nudging and Socially-Aware Conversational Strategies.* Front Robot AI, 2021. **8**: p. 733835.

10. Saravanan, A., et al. *Giving Social Robots a Conversational Memory for Motivational Experience Sharing*. in *2022 31st IEEE International Conference on Robot and Human Interactive Communication (RO-MAN)*. 2022.

11. Smriti, D., et al., *Motivational Interviewing Conversational Agent for Parents as Proxies for Their Children in Healthy Eating: Development and User Testing.* JMIR Hum Factors, 2022. **9**(4): p. e38908.
